# PBRM1 Regulates the Expression of Genes Involved in Metabolism and Cell Adhesion in Renal Clear Cell Carcinoma

**DOI:** 10.1371/journal.pone.0153718

**Published:** 2016-04-21

**Authors:** Basudev Chowdhury, Elizabeth G. Porter, Jane C. Stewart, Christina R. Ferreira, Matthew J. Schipma, Emily C. Dykhuizen

**Affiliations:** 1 Department of Medicinal Chemistry and Molecular Pharmacology, Purdue University, West Lafayette, Indiana, United States of America; 2 Metabolite Profiling Facility, Bindley Bioscience Center, Purdue University, West Lafayette, Indiana, United States of America; 3 NUSeq Core Facility, Center for Genetic Medicine, Northwestern University, Chicago, Illinois, United States of America; University of Kentucky College of Medicine, UNITED STATES

## Abstract

Polybromo-1 (PBRM1) is a component of the PBAF (Polybromo-associated-BRG1- or BRM-associated factors) chromatin remodeling complex and is the second most frequently mutated gene in clear-cell renal cell Carcinoma (ccRCC). Mutation of PBRM1 is believed to be an early event in carcinogenesis, however its function as a tumor suppressor is not understood. In this study, we have employed Next Generation Sequencing to profile the differentially expressed genes upon PBRM1 re-expression in a cellular model of ccRCC. PBRM1 re-expression led to upregulation of genes involved in cellular adhesion, carbohydrate metabolism, apoptotic process and response to hypoxia, and a downregulation of genes involved in different stages of cell division. The decrease in cellular proliferation upon PBRM1 re-expression was confirmed, validating the functional role of PBRM1 as a tumor suppressor in a cell-based model. In addition, we identified a role for PBRM1 in regulating metabolic pathways known to be important for driving ccRCC, including the regulation of hypoxia response genes, PI3K signaling, glucose uptake, and cholesterol homeostasis. Of particular novelty is the identification of cell adhesion as a major downstream process uniquely regulated by PBRM1 expression. Cytoskeletal reorganization was induced upon PBRM1 reexpression as evidenced from the increase in the number of cells displaying cortical actin, a hallmark of epithelial cells. Genes involved in cell adhesion featured prominently in our transcriptional dataset and overlapped with genes uniquely regulated by PBRM1 in clinical specimens of ccRCC. Genes involved in cell adhesion serve as tumor suppressor and maybe involved in inhibiting cell migration. Here we report for the first time genes linked to cell adhesion serve as downstream targets of PBRM1, and hope to lay the foundation of future studies focusing on the role of chromatin remodelers in bringing about these alterations during malignancies.

## Introduction

Kidney cancer is among the ten most common cancers in America, comprising approximately 62,000 new cancer cases and 14,000 deaths every year. Renal cell carcinoma (RCC) is the most common (~80%) and lethal type of kidney cancer in adults with clear cell RCC (ccRCC) as the most prevalent and aggressive subtype [1, 2]. ccRCC is named for its characteristic histological appearance caused by high glycogen and lipid content resulting from a glycolytic metabolic shift to a “Warburg effect”-like state [3]. Approximately 80% of ccRCCs have inactivation of VHL (von Hippel-Lindau), an E3 ubiquitin ligase involved in the degradation of hypoxia-inducible factor (HIF) transcription factors, HIF1α and HIF2α [4]. Although inheritance of VHL mutations causes a predisposition for ccRCC, deletion of VHL is not sufficient to cause cancer, and the loss of VHL alone provides neither prognostic nor therapeutic prediction values. Thus, other factors are required to drive ccRCC progression. In order to better understand genetic events causing ccRCC, exome sequencing of patient tumors has uncovered several novel genes significantly mutated in ccRCC, all of which encode for proteins that regulate chromatin. These novel genes include Polybromo-1 (PBRM1), BAP1, SETD2, KDM5C, and KDM6A. Polybromo-1 is the second most commonly mutated gene in ccRCC, with mutation rates at ~40% [5–9]. PBRM1 is a subunit of a subcomplex of the mammalian SWI/SNF (SWItch/Sucrose-NonFermentable) or BAF (BRG1 or BRM associated factors) chromatin remodeling complex termed PBAF (PBRM1-BAF). BAF complexes use energy from ATP to regulate transcription by altering chromatin structure and the placement of Polycomb across the genome. Subunits of the BAF complex are mutated in over 20% of human tumors [10, 11] yet the mechanisms involved in tumor suppression are still unclear.

Several studies have attempted to elucidate the molecular function of PBRM1 in ccRCC using transcriptional data from patient samples. While the panel of genes differentially regulated in *PBRM1-*mutated tumors express a hypoxic signature, no clear definition of PBRM1-regulated genes has come from these analyses, most likely due to the high heterogeneity of ccRCC tumors [9, 12, 13]. To overcome these obstacles, we report the development of isogenic ccRCC cell lines that permit dissecting the transcriptional role of PBRM1 in tumor suppression. We performed a comprehensive RNA-Seq analysis using a ccRCC cell line engineered with and without PBRM1 re-expression in order to specifically identify PBRM1 regulated genes. From this RNA-Seq dataset of PBRM1-regulated genes we identified several quintessential pathways involved in ccRCC oncogenesis including metabolism and hypoxia. In addition we identified many genes involved in cell adhesion that are uniquely regulated by PBRM1, and could be used to specifically characterize and treat PBRM1-mutated ccRCC tumors.

## Materials and Methods

### Cell culture

Caki-1 and Caki-2 cells (American Type Culture Collection, Manassas, VA) were cultured in McCoy’s 5A (Corning Mediatech, Inc., Manassas, VA) supplemented with 10% fetal bovine serum (Omega Scientific,Inc, Tarzana, CA), 1% antibiotics (100 U/ml penicillin and 100 μg/ml streptomycin; Corning Mediatech, Inc., Manassas, VA), 1% nonessential amino acids (Corning Mediatech, Inc., Manassas, VA) and 1% L-glutamine (Corning Mediatech, Inc., Manassas, VA) at 37°C in a humidified atmosphere in a 5% CO2 incubator. A704 and A498 cells (American Type Culture Collection, Manassas, VA) were cultured in Eagles Minimum Essential Media supplemented with 10% fetal bovine serum (Omega Scientific,Inc, Tarzana, CA), 1% antibiotics (100 U/ml penicillin and 100 μg/ml streptomycin; Corning Mediatech, Inc., Manassas, VA), 1% nonessential aminoacids (Corning Mediatech, Inc., Manassas, VA) and 1% L-glutamine (Corning Mediatech, Inc., Manassas, VA). All cells were maintained at 37°C in a humidified atmosphere in a 5% CO_2_ incubator.

### Constructs

PBRM1 knockdowns were performed using an empty pLKO.1 vector or pLKO.1 vector containing shRNA to human PBRM1 (TRCN0000015994, ThermoFisher Scientific, Waltham, MA). PBRM1 re-expression was performed by cloning full length PBRM1 from pBabepuroBAF180 (a gift from Ramon Parsons Addgene plasmid # 41078) into tet-inducible conditional lentiviral vector TetO-FUW (a gift from Rudolf Jaenisch Addgene plasmid # 20323), which was used with pLenti CMV rtTA3 Hygro (w785-1) (a gift from Eric Campeau Addgene plasmid # 26730) for tetracycline inducible expression.

### Lentiviral infection

HEK293T cells were transfected with lentiviral constructs along with lentiviral packaging vectors pMD2.G and psPAX2. After 48 h, supernatants were collected and virus isolated using ultracentrifugation at 20,000 r.p.m. for 2 h. Viral pellets were re-suspended in PBS and used to infect ccRCC cell lines by spinfection. Cells were selected with puromycin and/or hygromycin for one week to maintain stable lines. For inducible vectors, doxycycline was added to the culture media at 2 μg/mL for at least 72 hours prior to experiments.

### Sulforhodamine B assays

Sulforhodamine B assay was performed in 96-well plate format. Cells were fixed *in situ* by incubation with 50 μL of trichloroacetic acid at 4°C for 1 hour. After discarding the fixative solution, wells were rinsed thoroughly with tap water and air dried. Staining was performed by adding 50 μL of 0.4% Sulforhodamine B in 1% acetic acid solution to every well and the plate was incubated for 10 minutes at room temperature. Unbound Sulforhodamine was removed by washing the wells with 1% acetic acid. After air drying the plates, bound stain was solubilized with 10 mM Tris Base and the absorbance at a wavelength of 515 nm was read by Synergy 4 Hybrid Microplate Reader (BioTek, Winooski, VT).

### RNA isolation

Total RNA was extracted using TRIzol reagent (Life Technologies Corporation, Grand Island, NY) and cleaned up using RNeasy Mini Kit (Qiagen Inc., Valencia, CA) according to the manufacturer’s instructions.

### Library construction and sequencing

Library construction (100 bp, paired-end) and sequencing were carried out by Beijing Genomics Institute (BGI). The total RNA samples were enriched for mRNA by targeting polyadenylated (poly(A)) using oligo (dT) magnetic beads. Isolated mRNA was resuspended in fragmentation buffer and sonicated into short fragments of about 200 bp. mRNA was reverse transcribed into a single strand of causing random hexamer-primers. The second strand of cDNA was synthesized using DNA polymerase and the double stranded cDNA was purified with magnetic beads. End reparation and 3’-end Adenine addition were performed subsequently. Thereafter sequencing adaptors were ligated to the fragments and the fragments were enriched by PCR amplification. During the QC step, Agilent 2100 Bioanalyzer and ABI StepOnePlus Real-Time PCR System were used to qualify and quantify the sample libraries. Finally, the library products were sequenced on the Illumina HiSeq2000.

### Transcriptome analysis

The quality of DNA reads, in fastq format, was evaluated using FastQC. Adapters were trimmed, and reads of poor quality and those aligning to rRNA sequences were removed. The remaining clean reads were aligned to the human reference genome (hg19) using STAR [14]. Read counts for 25,369 genes were calculated using htseq-count [15] in conjunction with a standard gene annotation file for hg19 obtained from UCSC (University of California Santa Cruz; http://genome.ucsc.edu). Differential expression was determined using DESeq2 [16] using the counts from htseq-count as input (read counts pertaining to 25,369 genes in each of the samples). Built-in normalization algorithms of DESeq2 were used and an FDR-adjusted p-value of 0.05 used as the cutoff for determining differential gene expression. A pathway analysis was performed on gene lists using GeneCoDis [17–19] and Pre-ranked Gene Set Enrichment Analysis [20] to identify pathways enriched among genes that were upregulated and downregulated. Sequencing data for all the samples have been submitted at GEO (GSE76199). The data will be publicly available on acceptance of this manuscript for publication but currently reviewers can privately access the data by logging into their 'My NCBI' account and visiting http://www.ncbi.nlm.nih.gov/geo/query/acc.cgi?acc=GSE76199

### Real-time polymerase chain reaction (RT-PCR)

Total RNA was converted into cDNA using iScript Reverse Transcription Kit (Bio-Rad Laboratories, Inc., Hercules, California). All of the primers are listed in **S1 Table**. Real-time PCR was performed using a Bio-Rad CFX Connect Real-Time system and a Super Real Pre Mix Kit. The results were analyzed using the 2^(−ΔΔCT)^ comparative method. Each sample was tested in triplicate.

### Immunoblot analysis

Cells were lysed in cold Radio-Immunoprecipitation assay (RIPA) buffer containing freshly added protease inhibitor. The lysed cells were incubated on ice for 30 min and thereafter centrifuged at 14000 × g for 10 min at 4°C, and the supernatants were collected. Total protein was denatured for 10 min at 95°C, separated on a 10–15% SDS-polyacrylamide gel, and transferred to a PVDF membrane (Immobilon FL, EMD Millipore, Billerica,MA). The membrane was blocked with 5% bovine serum albumin (VWR, Batavia, IL) in PBS containing 0.1% Tween-20 (PBST) for 30 mins at room temperature and then incubated in primary antibodies overnight at 4°C. The primary antibodies used were directed against IGFBP3 (Santa Cruz Biotechnology Inc., Dallas, TX; sc-9028), Phospho-AKT (Ser473) (Cell Signaling Technology, Danvers, MA; 4060), Cleaved PARP (Asp214) (Cell Signaling Technology, Danvers, MA; 9541), AKT (Cell Signaling Technology, Danvers, MA; 9272), BRG1 (Abcam Plc, Cambridge,MA; ab110641), PBRM1 (Bethyl Laboratories, Montgomery, TX; A301-591A) and TBP (Abcam Plc, Cambridge,MA;ab818). The primary antibodies were detected by incubating the membranes in goat-anti-rabbit or goat-anti-mouse secondary antibodies (LI-COR Biotechnology, Lincoln, NE) conjugated to IRDye 800CW or IRDye 680 respectively for 1 h at room temperature, and the signals were visualized using Odyssey Clx imager (LI-COR Biotechnology, Lincoln, NE).

### Cell cycle distribution assays

Cells were harvested in the logarithmic growth phase and counted by MOXI Z Mini Automated Cell Counter (ORFLO Technologies, Ketchum, ID). 5 X 10^6^ cells were washed twice with ice cold PBS, resuspended in cold ethanol and incubated overnight at −20°C. The cells were centrifuged, and the supernatant was discarded. After the cells were washed twice with PBS, they were resuspended and stained with 50 μg/ml propidium iodide (PI), 100 μg/ml RNase A, and 0.2% Triton X-100. The samples were incubated in the dark at 4°C for 30 min and then analyzed via flow cytometry. Approximately 20,000 cells were examined per sample.

### Apoptotic assay

The percentage of cells undergoing apoptosis was determined using Annexin V-FITC/PI Apoptosis Detection Kit (BD Biosciences, Franklin Lakes, NJ). Cells were collected in logarithmic growth phase, washed twice with cold PBS, and adjusted to a density of 1 × 10^6^ cells/ml in binding buffer. The cell suspension (100 μl) was placed in a Falcon tube and was sequentially incubated in annexin V-FITC (5 μl) followed by PI (5 μl) at 20°C–25°C in the dark for 15 min. Then 400 μl binding buffer was added and the samples were analyzed by flow cytometry within one hour.

### Exposure to hypoxia

For hypoxia treatment, cells were plated 16 hours before placement in a modular incubator chamber (Billups-Rothenberg, Inc., Del Mar, CA) flushed with a gas mixture of 0.5% O_2_, 5% CO_2_ and 94.5% N_2_ (Indiana Oxygen Company, Indianapolis, IN) at 37˚C for 6 hours.

### Glucose uptake assay

Cells were plated (20,000 cells/well) in 96 well plates. After 16 hours, media from each well was aspirated and fresh media added (200μl/well). Fresh media was also added to wells not having cells and this served as control. After 6 hours of incubation, the media was collected and glucose content quantified using a glucose assay kit (Eton Bioscience, Inc., San Diego, CA) according to the manufacturer’s instructions. Glucose uptake was derived by subtracting the glucose content of media in wells having cells from those of wells not bearing cells (control). Glucose uptake rate (uM/hr/Absorbance) was finally calculated by dividing glucose uptake with time of treatment (6 hours) and relative number of cells (A_515nm_ determined by the Sulforhodamine assay).

### Imaging of actin cytoskeleton

Cells were plated on coverslips and grown until they achieved 70% confluency. The cells were washed twice with PBS and fixed in 3.7% methanol-free formaldehyde solution in PBS for 10 minutes at room temperature. The coverslips are rinsed thoroughly with PBS and permeabilized with 0.1% Triton X-100 in PBS for 5 minutes. The coverslips were blocked for 30 mins with PBS containing 1% BSA and then treated with 50 μL of Phalloidin conjugated to Texas Red (1:40; ThermoFisher Scientific, Waltham, MA) for 30 mins at Room Temperature. The coverslips were thoroughly rinsed with PBS containing 0.1% Tween 20, mounted on glass slides with Prolong Gold antifade containing DAPI (ThermoFisher Scientific, Waltham, MA) and imaged by Evos FL fluorescence microscope (ThermoFisher Scientific, Waltham, MA) as well as LSM 710 confocal microscope (Carl Zeiss Microscopy, LLC, Thornwood, NY). A total of five fields were counted for each condition (approximately 100–150 cells/field) by three independent observers in a blinded test. Each observer recorded the number of total cells, as determined by DAPI staining, as well as the number of cells with high concentrations of cortical actin staining as determined by Phalloidin staining. Finally, the percentage of cells with cortical F-actin in the two groups was determined and Fisher’s Exact Test was performed to evaluate the significance

### *In vitro* scratch test

Caki2+Vector and Caki2+PBRM1 cells were grown to 90% confluence in a 6 well plate. Scratches were made using a 1000μl pipet tip and the cells were rinsed gently with media to remove non-adherent cells. Fresh media was added to the cells and ten designated points on the scratch were imaged at 0h.The cells were allowed to incubate for 24 h and the designated points were imaged once again. ImageJ software was used to measure the average width (μm) of the scratch at 0h and 24 h and the cell migration/average closure rate was computed from the difference of the scratch width at 0h and 24h.

### Cholesteryl ester (CE) fingerprinting by mass spectrometry

Total lipid from the Caki2+Vector and Caki2+PBRM1 cells was extracted in accordance with the Bligh and Dyer protocol [21]. The chloroform phase containing lipids was dried under N_2_ stream and dissolved in 100μL of CHCl_3_/MeOH/H_2_O (vol/vol/vol proportion of 300/665/35) containing 300mM ammonium acetate. The volume of 8 μL of lipid extract was flow injected using a G1377A micro-autosampler into an ESI source of a Agilent 6410 triple-quadrupole mass spectrometer operated in positive ion mode for each MRM [586.2000 →369.1016 (CE12:0), 612.2000 → 369.1016 (CE 14:1), 614.2000 → 369.1016 (CE14:0), 640.6000 → 369.1016 (CE16:1), 642.6000 → 369.1016 (CE16:0), 664.6000 → 369.1016 (CE18:3), 666.6000 → 369.1016 (CE 18:2), 668.6000 → 369.1016 (CE18:1), 670.6000 → 369.1016 (CE18:0), 688.6000 → 369.1016 (CE20:5), 690.6000 → 369.1016 (CE20:4), 692.6000 → 369.1016 (CE20:3), 694.6000 → 369.1016 (CE20:2), 696.6000 → 369.1016 (CE20:1), 698.6000 → 369.1016 (CE20:0), 716.6000 → 369.1016 (CE22:6), 718.6000 → 369.1016 (CE22:5), 720.6000 → 369.1016 (CE22:4), 722.6000 → 369.1016 (CE22:2) and 724.6000 → 369.1016 (CE22:1)] [22, 23]. Instrument’s dwell time was 20ms and collision energy 15. Data was collected for 1 min, in which 106 scans per CE species have been obtained. Data was processed for chronogram smoothing and subsequently the CE peak list was generated using Agilent Mass Hunter B.06.00 software. The proportion of CE species in every sample was obtained by dividing the total ion intensity (sum of 106 scans) for each CE species by the total ion intensity obtained for all CE species assayed [24]. A semi-quantitative comparison of the proportion of CE species in Caki2+PBRM1 and Caki2+Vector was performed and CE species displaying significant (Student’s T-test p-value <0.05) differences between the two groups were plotted.

## Results & Discussion

### PBRM1 expression in RCCs

**Fig 1:**
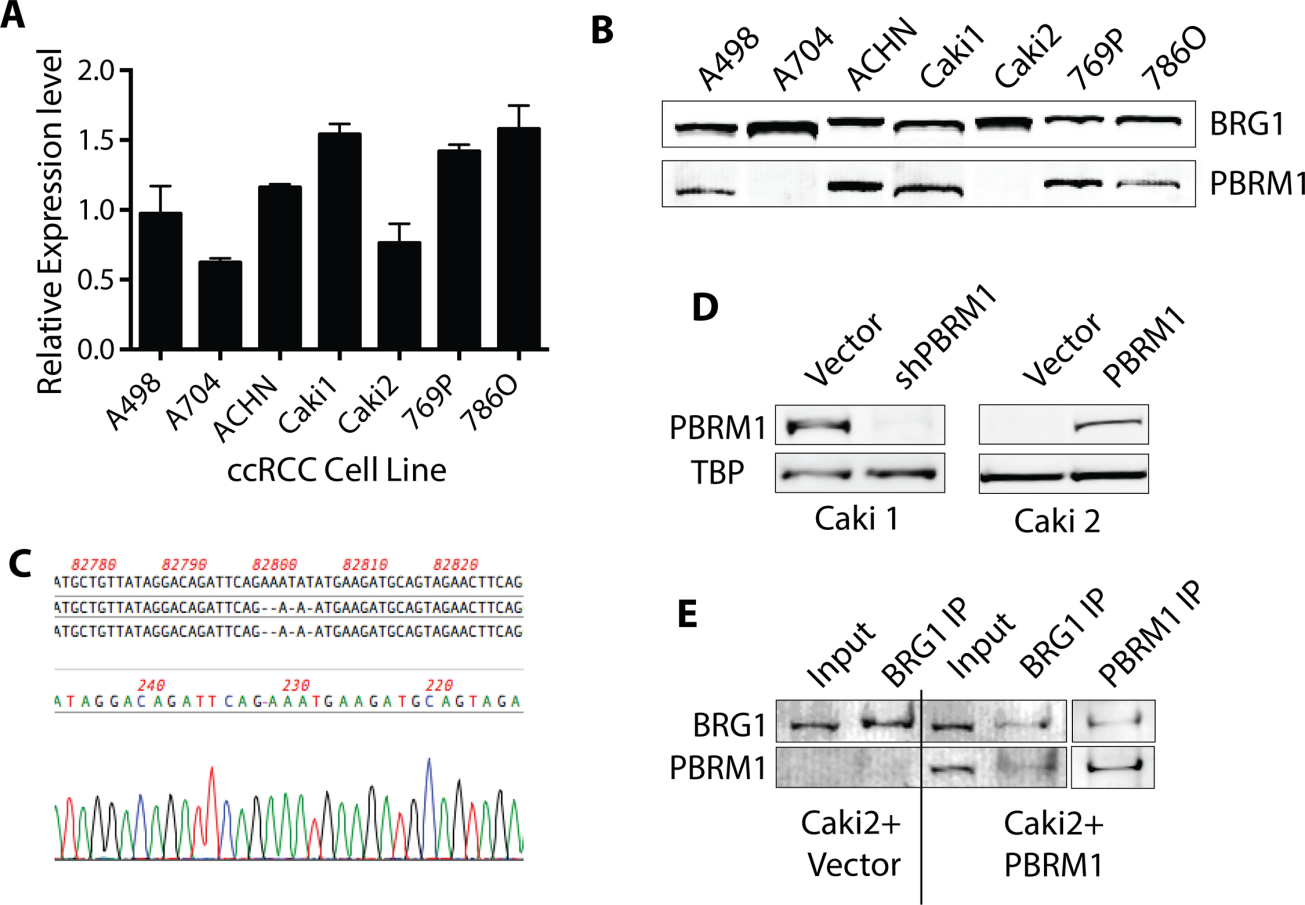
Characterization of PBRM1 in ccRCC cells. (A) Relative transcript expression of PBRM1 in ccRCC cell lines. (B) Protein levels of BRG1 and PBRM1 in ccRCC cell lines. (C) Characterization of deletion in exon 17 of PBRM1 gene in Caki2 cells. (D) Protein levels of PBRM1 in isogenic ccRCC cells (Caki1 with PBRM1 knockdown and Caki2 with PBRM1 re-expression). (D) BRG1 and PBRM1 blotting in BRG1 or PBRM1 immunoprecipitates of Caki2+Vector and Caki2+PBRM1.

In order to identify ccRCC cell lines with PBRM1 loss, we obtained a panel of ccRCC cell lines from ATCC and tested them for PBRM1 mRNA and protein expression. As depicted in **Fig 1A**, all of the cell lines express mRNA for PBRM1 using primers that span the exon 22/23 junction [6]. This is in accordance with reports from human tumors that report loss of PBRM1 protein function through mutation, not transcriptional silencing [7]. Using immunoblot analysis (**Fig 1B**), we determined that the cell lines 786O, 769P, Caki1, and A498 express PBRM1 in accordance with reports of being genetically wild type, ACHN expresses PBRM1 and has a heterozygous nonsense mutation [25], and the cell lines A704 and Caki2 do not express PBRM1, as previously reported [12]. The loss-of-function gene mutations have been identified for A704 [25] but to the best of our knowledge, the sequencing of the PBRM1 gene in Caki2 has not been previously reported. Using Sanger sequencing, we identified a previously uncharacterized 4 bp deletion in exon 17, accounting for the loss of protein expression in this cell line (**Fig 1C**).

### Generation of Isogenic cell lines for controlled studies of PBRM1 function

We created complementary cell lines using lentiviral knockdown of PBRM1 in A498 and Caki1 and the re-expression of PBRM1 in A704 and Caki2 using lentiviral re-expression of PBRM1 with a tet-inducible vector (**Fig 1D**). We confirmed reincorporation of PBRM1 into the BAF complex using immunoprecipitation followed by immunoblot analysis (**Fig 1E**). In addition, we used glycerol gradient analysis to confirm that PBRM1 is fully reincorporated specifically into the PBAF complex and that overexpression doesn’t result in PBRM1 monomer or alterations in PBAF complex stoichiometry (**S1 Fig**). We did not observe any large scale destabilization or re-organization of the BAF or PBAF complexes in Caki2 cells or in Caki1 cells upon PBRM1 knockdown, although we did observe a reproducible increase in ARID2 protein levels with decreased PBRM1, which may play a role in altering PBAF-mediated transcription in ccRCC with PBRM1 mutations.

### Effect of PBRM1 expression on cell proliferation

**Fig 2:**
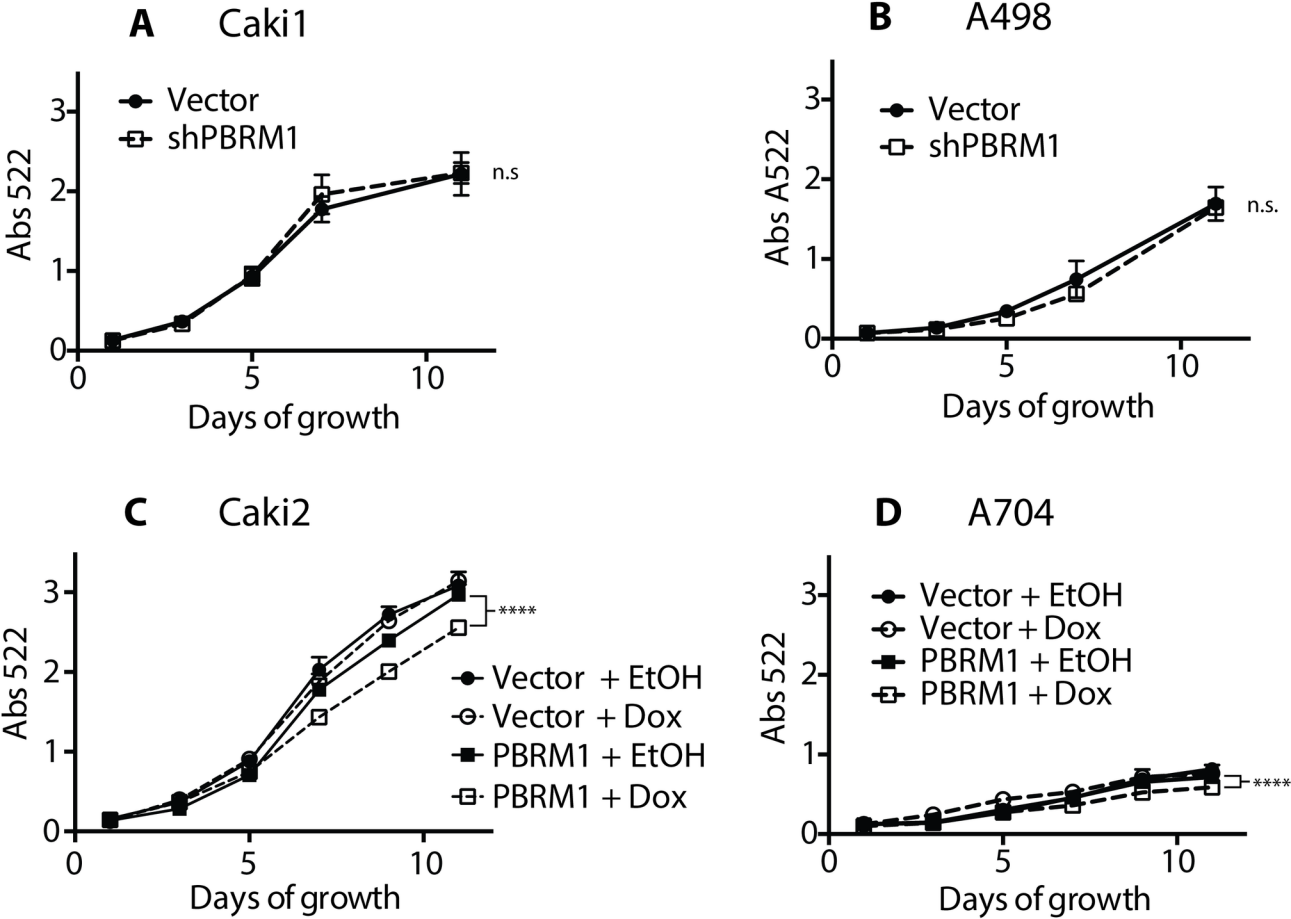
Presence of PBRM1 resulted in decreased proliferation rate of ccRCC cells. Cellular proliferation rate was monitored in (A) Caki1 and (B) A498 with either control knockdown or PBRM1 knockdown, as well as (C) Caki2 and (D) A704 with either control vectors or PBRM1 re-expression vectors. Both cell lines were treated with ethanol or doxycycline to control for cell line variation or doxycycline effects. n = 8 independent biological replicate experiments. P < 0.0001 (****) (two-way ANOVA test) between PBRM1 re-expression cell lines treated with ethanol (EtOH) or Doxycycline (Dox) at day 11. Error bars represent s.e.m.

We performed proliferation analysis with PBRM1 knockdown in Caki1 cells and A498 cells and were surprised to find no significant effect on proliferation under standard cell culture conditions (**Fig 2A and 2B**). We hypothesized that due to the heterogeneity of mutations in ccRCC, PBRM1 may serve a redundant role in tumor suppression with other mutated genes in these ccRCC cell lines. Thus we focused on cell lines with PBRM1 mutations with the assumption that loss of PBRM1 acts as a driver in these cancers. Although the effects of re-expressing PBRM1 in Caki2 and A704 were modest, we observed a definitive role of PBRM1 reexpression in reducing cellular proliferation under standard cell culture conditions (**Fig 2C and 2D**). Due to the low proliferative rate of the A704 cell line, we pursued the isogenic Caki2 cell lines for further investigation of the role of PBRM1 on gene transcription.

### Differentially expressed genes upon PBRM1 re-expression in Caki-2

**Fig 3:**
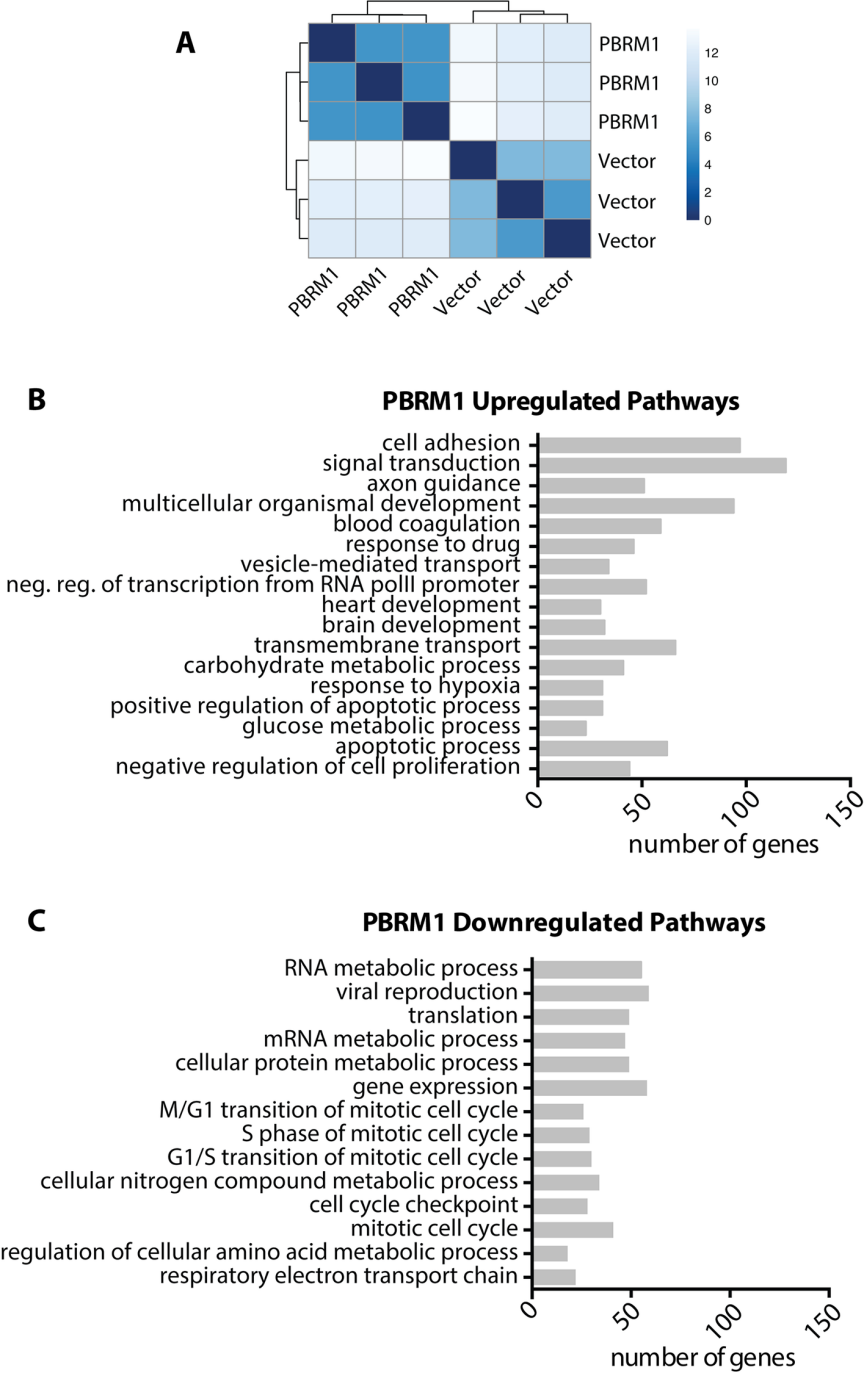
Summary of differentially expressed genes in Caki2 cells upon PBRM1 reexpression. (A) Hierarchical Cluster Dendrogram of Caki2+Vector and Caki2+PBRM1 samples sequenced. (B) GO Biological Processes enriched by genes upregulated upon PBRM1 reexpression. (C) GO Biological Processes enriched by genes downregulated upon PBRM1 reexpression.

To identify key pathways influenced by PBRM1 activity, we performed an RNA-Seq analysis of Caki2 cells with and without PBRM1 re-expression (Caki2+Vector and Caki2+PBRM1). Hierarchical clustering with un-normalized counts as well as normalized regularized-logarithm transformed count data demonstrated similarities between the biological replicates illustrated by Euclidean distances (**Fig 3A**). DESeq2 analysis, comparing the transcript expression of Caki2+PBRM1 and Caki2+Vector cells revealed that 2,464 genes were differentially expressed (FDR-padj<0.05) (**S2 Table**). Of these, 65 were upregulated and 6 were downregulated more than two fold in Caki2+PBRM1 compared to Caki2+Vector cells. 97% of the differentially expressed genes (n = 2,393) underwent mild to moderate (0–2 log_2_ fold) levels of differential expression upon PBRM1 re-expression. GO analysis established that upregulated genes enriched biological processes such as cell adhesion (GO:0007155), apoptotic process (GO:0043065), negative regulation of cell proliferation (GO:0008285), carbohydrate metabolic process (GO:0005975) and response to hypoxia (GO:0001666) among others (**Fig 3B and S2 Table**) and downregulation of genes involved in mitotic cell cycle (GO:0000278), G1/S transition (GO:0000082) and metabolic processes (GO:0044267) (**Fig 3C and S2 Table**).

### PBRM1 in cell cycle and apoptosis

**Fig 4:**
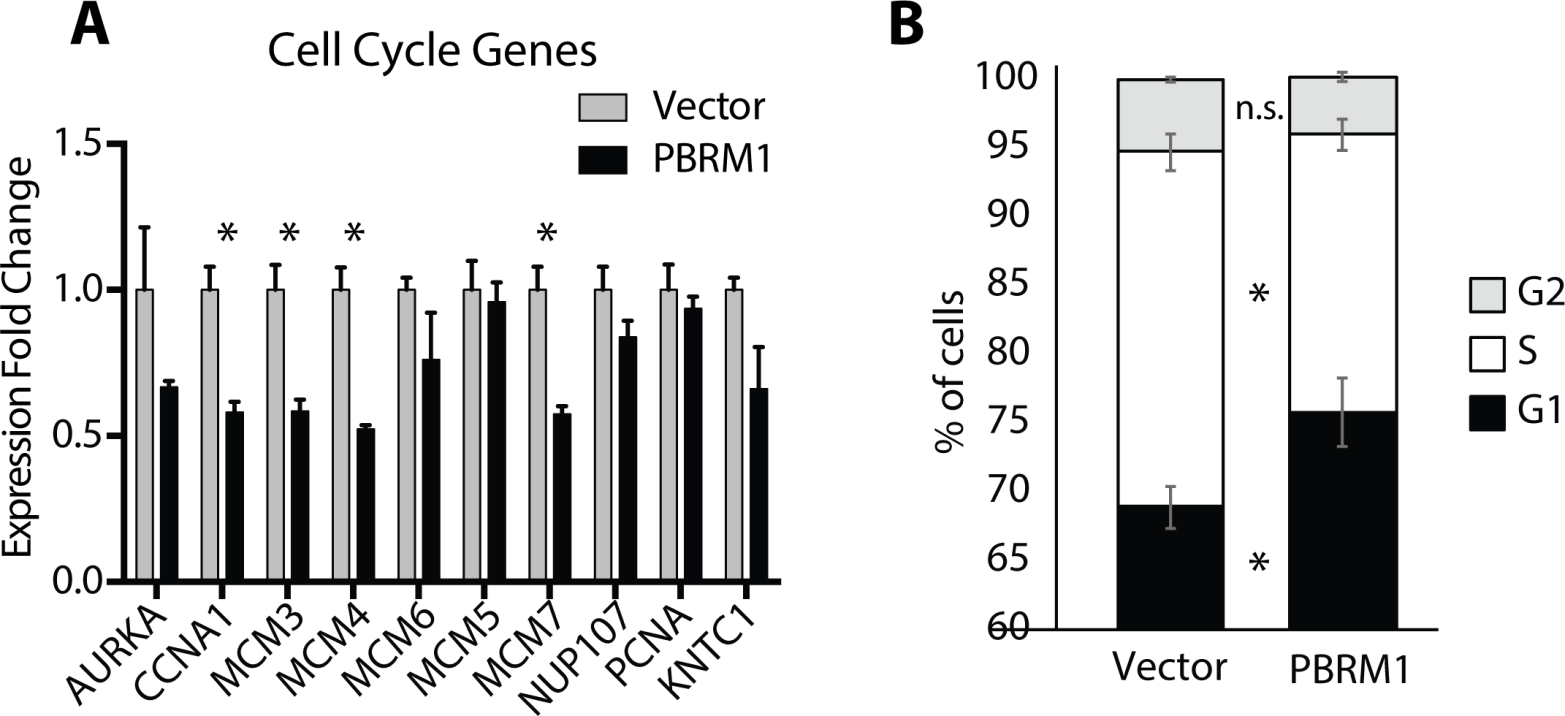
Downregulation of genes involved in cell proliferation upon PBRM1 re-expression. (A) Validating relative expression of genes regulating cell cycle (identified in RNA_seq data) in Caki2+Vectorand Caki2+PBRM1 cells by qRTPCR. A designation of * = P < 0.05 (paired Student *t*-test). n = 3 independent biological replicate experiments. Error bars represent s.e.m. (B) Percentage of cells in different stages of cell cycle (G1, S and G2) determined by flow cytometric analysis. A designation of * = P < 0.05 (paired Student *t*-test). n = 3 independent biological replicate experiments. Error bars represent s.e.m.

In accordance with inhibition of proliferation observed upon PBRM1 reexpression (**Fig 2C**), RNA-seq analysis revealed downregulation of genes involved in cell cycle including 30 genes associated with G1/S transition (**S2 Table**) and qRTPCR confirmed the downregulation of several representative genes (**Fig 4A**). Flow cytometric analysis demonstrated a role for PBRM1 in G1/G0 arrest with a significant reduction in S-phase cells upon PBRM1 re-expression in Caki2 cells (**Fig 4B**). This is similar to the cell cycle effects observed upon PBRM1 re-expression in PBRM1 mutant breast cancer [26]. To further define the G1/G0 arrest observed with PBRM1 re-expression we wished to determine whether increased apoptosis contributes to the G1/G0 cell cycle block. From the transcriptional analysis, we observed upregulation of both pro-apoptotic and anti-apoptotic genes (**S2 Table**) and validated some of them by qRTPCR (S2 **Fig**). Low levels of apoptotic markers Annexin V and cleaved PARP [27], in both Caki2+Vector and Caki2+PBRM1 cells led us to conclude the G1/G0 cell cycle block observed for the Caki2+PBRM1 cell line did not result in increased apoptosis (S2 **Fig**).

### PBRM1 in hypoxia

ccRCC is characterized by a metabolic shift mediated through altered hypoxic signaling. PBRM1-deficient tumors in particular display a hypoxic transcriptional signature, possibly implying that deletion of PBRM1 acts in concert with, or facilitates, pro-oncogenic hypoxic pathways [6, 28], In apparent contradiction to this, we observe hypoxia-response genes as significantly upregulated upon PBRM1 re-expression. This is in agreement with the observation that SWI/SNF subunits BRG1, BAF155, and BAF57 are required for the induction of HIF1α target genes in other cell types [29, 30]. The diversity of HIF1α and HIF2α aberrations in ccRCC makes it challenging to evaluate their individual contribution to supporting ccRCC; however, Shen et al have documented the enhancement and inhibition of cell growth upon knocking down HIF1α and HIF2α respectively in the Caki2 cells as well as in mouse xenografts supporting the hypothesis that HIF1α itself acts as a tumor suppressor and is often deleted in renal cancer cell lines, while HIF2α is the primary oncogenic driver [31–33]. Therefore, it follows that PBRM1 may act as a tumor suppressor by facilitating the expression of HIF1α but not HIF2α target genes.

**Fig 5:**
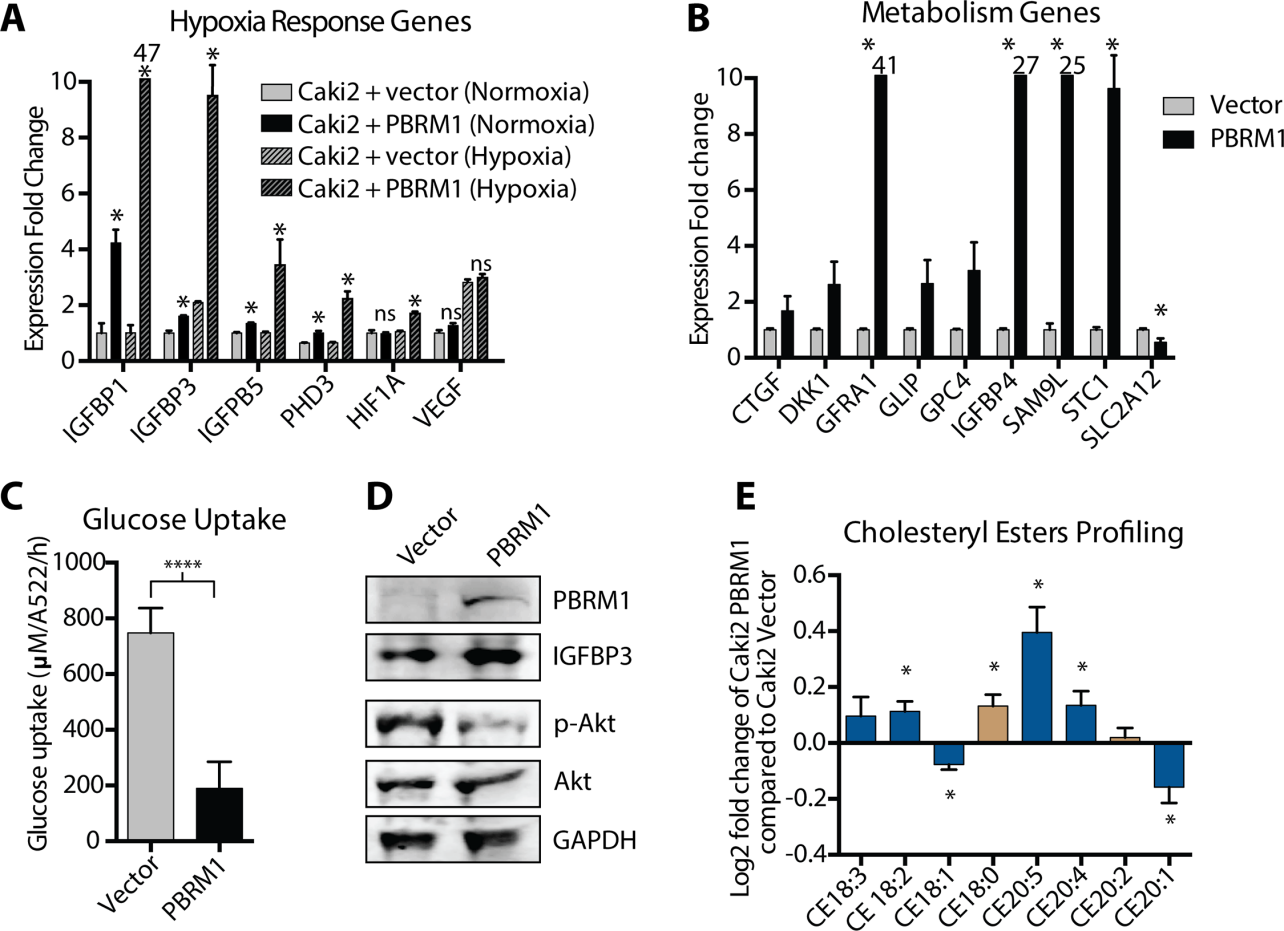
Alteration of metabolism upon PBRM1 re-expression. (A) Relative expression of genes regulating hypoxic response in Caki2+Vectorand Caki2+PBRM1 cells subjected to normoxic and hypoxic (0.5% O_2_) conditions. A designation of * indicates p < 0.05 (Student *t*-test). n = 3 independent biological replicate experiments. Error bars represent s.e.m. (B) Validating relative expression of genes regulating primary metabolic processes (identified in RNA_seq data) in Caki2+Vectorand Caki2+PBRM1 cells by qRTPCR. (C) Determination of glucose uptake in Caki2+Vectorand Caki2+PBRM1 cells. A designation of **** indicates p < 0.0001 (paired Student *t*-test). n = 5 independent biological replicate experiments. Error bars represent s.e.m. (D) Immunoblot of IGFBP3, phosphorylated AKT and unphosphorylated AKT in Caki2+Vectorand Caki2+PBRM1 cells. (E) Semi-quantitative estimation of alteration of cholesteryl esters in Caki2+PBRM1 compared to Caki2-Ø. Bars depicted in blue reverse the differences observed during the progression from normal kidney epithelium to ccRCC and bars depicted in tan compound those differences. A designation of * = P < 0.05 (paired Student *t*-test). n = 3 independent biological replicate experiments. Error bars represent s.e.m.

Consequently, we explored the changes in gene expression of IGBP1, PHD3 and HIF1α, which are upregulated in Caki2+PBRM1. IGFBP1 is a member of the IGFBP family, which are HIF target genes that play important roles in regulating glucose availability by binding to insulin-like growth factors. Under normoxia, IGFBP1 was upregulated upon PBRM1 reexpression (**Fig 5A)**, and hypoxia induced dramatic increase in the levels of more than 4 fold for IGFBP1 (and also other members of the IGFBP family like IGFBP3 and IGFB5) in Caki2+PBRM1. In contrast, hypoxic exposure did not bring about any change in levels of IGFBP1 or the other members of the IGFBP family in Caki2+Vector cells. HIF1α and PHD3 are canonical HIF targets, and exposure to hypoxia resulted in increase of steady state levels of HIF1α and PHD3 in Caki2+PBRM1 but not in Caki2+Vector. On the other hand, hypoxia induced an increase in VEGF expression (a classic oncogenic HIF target gene, widely used in studying hypoxia response and not implicated in our RNA-seq data) in both Caki2+PBRM1 and Caki2+Vector cells, driving home the point that PBRM1 may regulate the expression of a subset of HIF target genes, possibly distinguishing between HIF1α and HIF2α targets [32].

### PBRM1 in glycolysis and cholesterol homeostasis

One of the defining features of ccRCC is the increase in glycolysis and activation of the PI3K signaling pathway [7]. Many of the PBRM1 upregulated genes, including the hypoxia-response genes, have been shown to decrease PI3K signaling through the inhibition of insulin receptor signaling and glycolysis [7, 34]. These genes code for glycolytic enzymes, solute carriers, and proteins involved in cholesterol homeostasis. We identified genes upregulated by PBRM1 that inhibit glycolysis, as well as several genes downregulated by PBRM1 involved in facilitating glycolysis, and confirmed the expression several of them using qRTPCR (**Fig 5B**). In accordance with this result we observed a significant decrease in glucose uptake (p = 1.2e-5) upon PBRM1 re-expression (**Fig 5C)** as well as a decrease in PI3K signaling using immunoblot analysis of AKT phosphorylation (**Fig 5D**)

The histological appearance of lipid accumulation in ccRCC is well established and leads to the name “clear cell” renal carcinoma [35, 36]. Among all lipid classes, Cholesteryl-esters illustrate the most dramatic alterations in their profile in ccRCC [35, 37]. Though the precise molecular mechanism is unknown, the increased accumulation in cholesteryl oleate (CE18:1) and decreased accumulation in cholesterol linoleate (CE18:2) has been characterized in ccRCC [35, 37]. Upon PBRM1 re-expression in Caki2 cells, we observed a decrease in CE18:1 and an increase in CE18:2 which was in agreement to previous studies (**Fig 5E**) [35, 37]. Additionally we observed the increased representation of CE16:0, CE20:5, CE20:4 and decreased representation of CE20:1 in comparing the lipid profile of Caki2+PBRM1 with that of Caki2+Vector, which was consistent with the comparison of normal kidney tissue and renal cell carcinoma obtained from nephrectomies from six patients reported earlier [37]. The increased representation of CE20:3 and CE22:4 upon PBRM1 re-expression in Caki2 cells was not consistent with the profile of normal kidney tissue in the earlier study [37]. We are reporting the novel observation of the increased representation of CE16:0, CE18:1, CE24:0, CE22:0 and CE22:4 upon PBRM1 re-expression, which have not be quantified in any study to the best of our knowledge (**S3 Fig**).

### PBRM1 in cell adhesion

**Fig 6:**
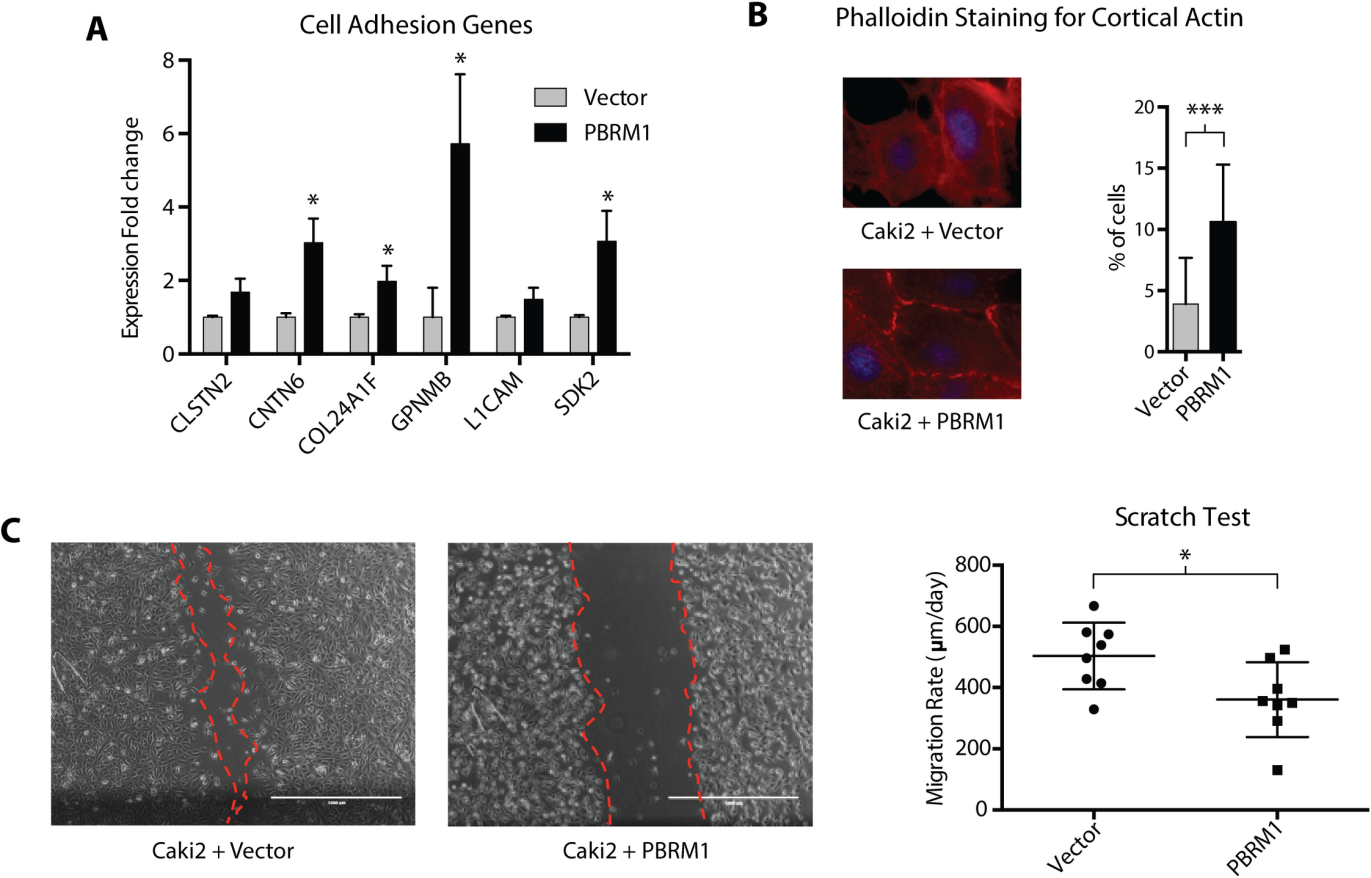
Alteration of cell-cell adhesion upon PBRM1-reexpression. (A) Validating relative expression of genes regulating cell-cell adhesion (identified in RNA_seq data) in Caki2+Vectorand Caki2+PBRM1 cells by qRTPCR. A designation of * indicates p < 0.05 (Student *t*-test). n = 3 independent biological replicate experiments. Error bars represent s.e.m. (B) Phalloidin staining of F actin (red channel) and DAPI staining of nucleus (blue channel) in Caki2+Vectorand Caki2+PBRM1 cells. A designation of *** indicates p < 0.001 (Fisher’s exact *t*-test). n = 5 independent biological replicate experiments. Error bars represent s.e.m. (C) *In vitro* scratch assay illustrating cell migration 24 hours after scratch wound inflection. A designation of * indicates p < 0.05 (Student *t*-test). n = 8 independent biological replicate experiments. Error bars represent s.e.m.

Cell adhesion is essential in adherent cells serving as molecular scaffolds linking the cellular cytoskeleton to the extracellular environment [38]. Normal and non-metastasizing cells are observed to form close focal contacts while metastasizing cells maintain limited areas of close contact [39], suggesting that aberrations in cell adhesion may be responsible for certain aspects of malignant cell behavior [40]. The relationship between cell adhesion and actin cytoskeleton is intertwined as the dynamic equilibrium of actin polymerization and disassembly dictate the type and function of the resultant adhesions complexes[41]. qRTPCR of representative genes involved in cell adhesion confirmed the upregulation upon PBRM1 reexpression as determined by RNA-seq (**Fig 6A**). Immunofluorescence of F-actin demonstrated a significant increase in the percentage of cells with cortical actin cytoskeleton in Caki2+PBRM1 with respect to Caki2+Vector (**Fig 6B** and **S4 Fig**). The “cortical actin cytoskeleton” phenotype, a hallmark of epithelial cells [42, 43] is believed to promote cell-cell contact and act as an important player in cellular morphogenesis [44–46]. In cancers of epithelial cell origin, the cortical actin cytoskeleton is disrupted and subsequently reorganized to facilitate cell migration and invasion [47–50]. Reexpression of PBRM1 also caused a significant inhibition of cell migration in an *in vitro* scratch test [51] (**Fig 6C**), further validating PBRM1 in the regulation of cell adhesion and the maintenance of a more epithelial cell state.

### Differentially expressed genes in PBRM1 mutant tumors derived from patient samples

**Fig 7:**
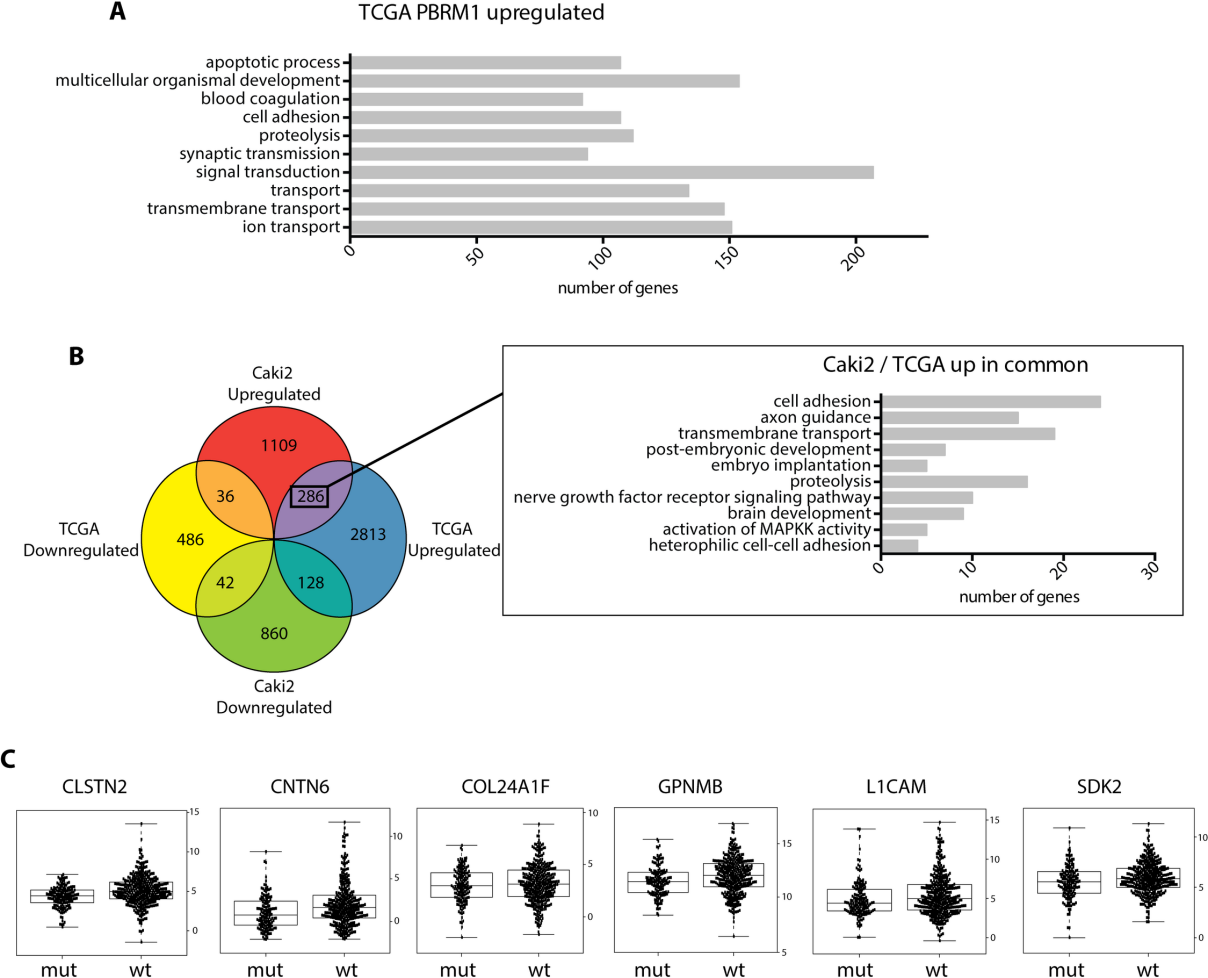
Comparative analysis of genes with differential expression in the presence of PBRM1 between ccRCC cells and ccRCC TCGA biospecimen. (A) GO Biological Processes enriched by genes upregulated in TCGA ccRCC biospecimen with no PBRM1 mutation compared to ccRCC biospecimen with PBRM1 mutations. (B)Venn diagram illustrating the number of differentially expressed genes overlapping between the 2 datasets (Genes with differential expression in 1. Caki2+PBRM1 with respect to Caki2+Vector and 2. ccRCC biospecimen with no PBRM1 mutation with respect to biospecimen with PBRM1 mutations). Inset illustrates GO Biological Processes enriched by upregulated genes in both (1) PBRM1 reexpressed Caki2 cells and (2) TCGA ccRCC biospecimen with no PBRM1 mutation. (C) Box Plot showing the relative expression (log_2_ RSEM) of genes involved in cell adhesion in TCGA biospecimen.

In order to further investigate the set of PBRM1 induced differentially expressed genes that may be clinically relevant, we compared the transcription profile of 499 ccRCC specimens deposited at TCGA. 354/499 specimens are reported to have no mutation in PBRM1 gene while the remaining 145 have mutations in the PBRM1 gene (**S2 Table, Fig 7A** and **S5 Fig**). A comparison between the transcription profile in (i) ccRCC patients with and without mutated PBRM1 and (ii) Caki2 cells with and without PBRM1 were originally envisioned to identify clinically relevant transcriptional targets of PBRM1. A major caveat, however, is that ccRCC is a heterogeneous cancer characterized by a metabolic shift [52] but not absolutely defined by a particular mutation or set of mutations. Therefore, ccRCC patients without mutated PBRM1 may have alterations in different genes that affect the same pathways, making the mutational status of PBRM1 irrelevant. Therefore, pathways uniquely regulated by PBRM1 in patient samples don’t include classical metabolic pathways known to be universally deregulated in ccRCC (**Fig 7A**, **S5 Fig**). Of the genes co-regulated in patient samples and Caki2 cells, 286 were upregulated and only 42 were downregulated (FDR adjusted p <0.05). The upregulated genes were involved in biological processes like cell adhesion (GO:0007155; GO:0007157; GO:0022409) and actin cytoskeleton organization (GO:0030036) indicating a unique role for PBRM1 in the expression of these genes that is not redundant with other tumor suppressor genes commonly mutated in ccRCC (**Fig 7B** and **S3 Table**). The changes in expression in ccRCC patients of representative genes involved in cell adhesion that were also altered upon PBRM1 re-expression in Caki2 cells were validated by qRTPCR (**Fig 7C**).

## Conclusion

ccRCC is a metabolic cancer characterized by a shift to aerobic glycolysis facilitated by alterations in hypoxia transcriptional pathways. Fundamental pathways driving ccRCC (and therapeutics targeting these pathways) have been elucidated as a result of deciphering the mechanism of VHL, the most commonly mutated gene in ccRCC. Still, the loss of VHL and increase in HIF transcriptional activity is not sufficient to cause or drive cancer, indicating that additional factors are required for promoting oncogenesis, including the mutation of epigenetic regulators such as PBRM1. Using a ccRCC with a loss-of-function PBRM1 mutation, we found that re-expression of PBRM1 reverses some of the metabolic effects characteristic of ccRCC, including increased glucose uptake, shifts in cholesterol esters, and increased insulin/PI3K signaling. In addition, we have found a unique role for PBRM1 in regulating cell adhesion genes as confirmed by co-occurrence in ccRCC tumors with PBRM1 mutation. Further work will be needed to determine whether this gene signature truly represents increased metastatic potential in PBRM1-mutant ccRCC, and ultimately, if this gene signature can be used to design or predict effective therapeutics.

PBRM1 is a subunit of the SWI/SNF chromatin remodeling complex, which has been found to be involved in developmental transitions and the maintenance of cell-type specific transcriptional programs. It acts in conjunction with transcription factors and can be recruited in response to signaling; for PBRM1 this recruitment is likely tied to patterns of histone acetylation that define certain gene targets. PBRM1 upregulated genes include targets of retinoic acid receptor [53, 54] and FOXO4 [55, 56] binding, in addition to HIF1α binding, all of which have been implicated in antagonizing ccRCC progression. To mechanistically understand the relevance of the downstream transcriptional targets we identified in ccRCC, the next step will be to define PBRM1 binding across the genome and determine cooperating transcription factors. This will further our understanding of ccRCC progression and uncover new therapeutic targets of PBRM1-mutated ccRCC.

## Supporting Information

S1 Fig Glycerol gradient analysis of BAF/PBAF complexes in Caki1 and Caki2 cell lines. (A) In Caki1 cells PBAF (represented by PBRM1 and ARID2) elutes in higher fractions of a glycerol gradient indicating a larger size than BAF (represented by SNF5, which exists in both BAF and the less abundant PBAF), (B) Upon PBRM1 knockdown, the size of PBAF decreases, as represented by a shift in ARID2 staining to earlier fractions (C) In Caki2 cells with PBRM1 mutation, ARID2 staining is detected in an earlier fraction. (D) Upon the re-expression of PBRM1, the size of PBAF (represented by ARID2 staining) increases and elutes in later fractions, mimicking the staining profile observed for Caki1 cells.
(TIF)

S2 Fig (A) Validating relative expression of genes regulating apoptosis (identified in RNA_seq data) in Caki2+Vector and Caki2+PBRM1 cells by qRTPCR. A designation of * indicates p < 0.05 (Student *t*-test). n = 3 independent biological replicate experiments. Error bars represent s.e.m. (B) Blot of cleaved PARP (apoptotic marker) and (C) Percentage of apoptotic cells determined by flow cytometric analysis on Caki2+Vectorand Caki2+PBRM1 cells. n = 3 independent biological replicates. Error bars represent s.e.m.
(TIF)

S3 Fig Semi-quantitative analysis of CE alterations upon PBRM1 reexpression. The log2 Fold change, SEM and p value (Student’s T-test) of the analyzed CEs. n = 3 biological replicates.
(TIF)

S4 Fig Alteration of actin cytoskeleton reorganization upon PBRM1 reexpression. (A) Representative Field showing Phalloidin (F-actin) and DAPI (nucleus) staining in Caki2+Vector and Caki2+PBRM1 cells used for unbiased quantification of cells with high cortical actin. 4X magnified image of this is shown in Fig 6B. (B) Examples of confocal images of Caki2+Vector and Caki2+PBRM1 cells stained with Phalloidin. Scale bar depicts 10 µm.
(TIF)

S5 Fig Biological Pathways enriched by down regulated genes in the presence of wt PBRM1 in ccRCC tumor samples. GO Biological Processes enriched by genes downregulated in TCGA ccRCC biospecimen with no PBRM1 mutation compared to ccRCC biospecimen with PBRM1 mutations.
(TIF)

S1 Table List of Primers used in qPCR. The sequence of primers of the respective genes used in our study.
(XLS)

S2 Table List of Differentially Expressed Genes upon PBRM1 reexpression in Caki2. (Tab 1-DEGS) The Differentially expressed genes (DEGs) in Caki2+PBRM1 cells compared to Caki2+Vector. The columns represent (i) Gene Symbols of DEGs, (ii) Description of the DEGs, (iii) Log 2 Fold Change, (iv) BH–FDR-adjusted pvalue and (v) whether the fold change of DEG is significant. (Tab 2-Upregulated_Genes_GO (BP)) GO (BP) terms enriched by upregulated genes in Caki2+PBRM1. The columns represent (i) GO terms enriched, (ii) Description of the GO terms, (iii) the number of DEGs enriched in the GO term, (iv) p-value associated with enrichment and (v) Gene Symbols of the DEGS enriched in the GO term. (Tab 3-Downregulated_Genes_GO (BP)) GO (BP) terms enriched by downregulated genes in Caki2+PBRM1. The description of the columns are same as the ones in Tab 2.
(XLS)

S3 Table List of Differentially Expressed Genes in TCGA ccRCC with no PBRM1 mutations. (Tab 1-TCGA normal PBRM1 Vs Mutated) The Differentially expressed genes (DEGs) in ccRCC biospecimen with no PBRM1 mutation compared to ccRCC biospecimen with PBRM1 mutations. The columns represent (i) Gene Symbols of DEGs, (ii) Log 2 Fold Change and (iii) BH–FDR-adjusted pvalue. (Tab 2-Upregulated_Genes_GO (BP)) GO (BP) terms enriched by upregulated genes in ccRCC with no PBRM1 mutations. The columns represent (i) GO terms enriched, (ii) Description of the GO terms, (iii) the number of DEGs enriched in the GO term, (iv) p-value associated with enrichment and (v) Gene Symbols of the DEGS enriched in the GO term. (Tab 3-Downregulated_Genes_GO (BP)) GO (BP) terms enriched by downregulated genes in ccRCC with no PBRM1 mutations. The description of the columns are same as the ones in Tab 2.
(XLS)

S4 Table GO (BP) terms enriched by Differentially Expressed Genes in the presence of PBRM1 both in Caki2 cells and TCGA ccRCC biospecimen. (Tab 1-Upregulated_Genes_GO (BP)) GO (BP) terms enriched by upregulated genes in Caki2+PBRM1 and ccRCC biospecimen with no PBRM1 mutations. The columns represent (i) GO terms enriched, (ii) Description of the GO terms, (iii) the number of DEGs enriched in the GO term, (iv) p-value associated with enrichment and (v) Gene Symbols of the DEGS enriched in the GO term. (Tab 2-Downregulated_Genes_GO (BP)) GO (BP) terms enriched by downregulated genes in Caki2+PBRM1 and ccRCC biospecimen with no PBRM1 mutations. The description of the columns are same as the ones in Tab 1.
(XLS)
