# Supplementary figures and images for: PBRM1 Regulates the Expression of Genes Involved in Metabolism and Cell Adhesion in Renal Clear Cell Carcinoma
Source: PLoS One. 2016 Apr 21;11(4):e0153718. doi: 10.1371/journal.pone.0153718 (PMC4839679; doi:10.1371/journal.pone.0153718)

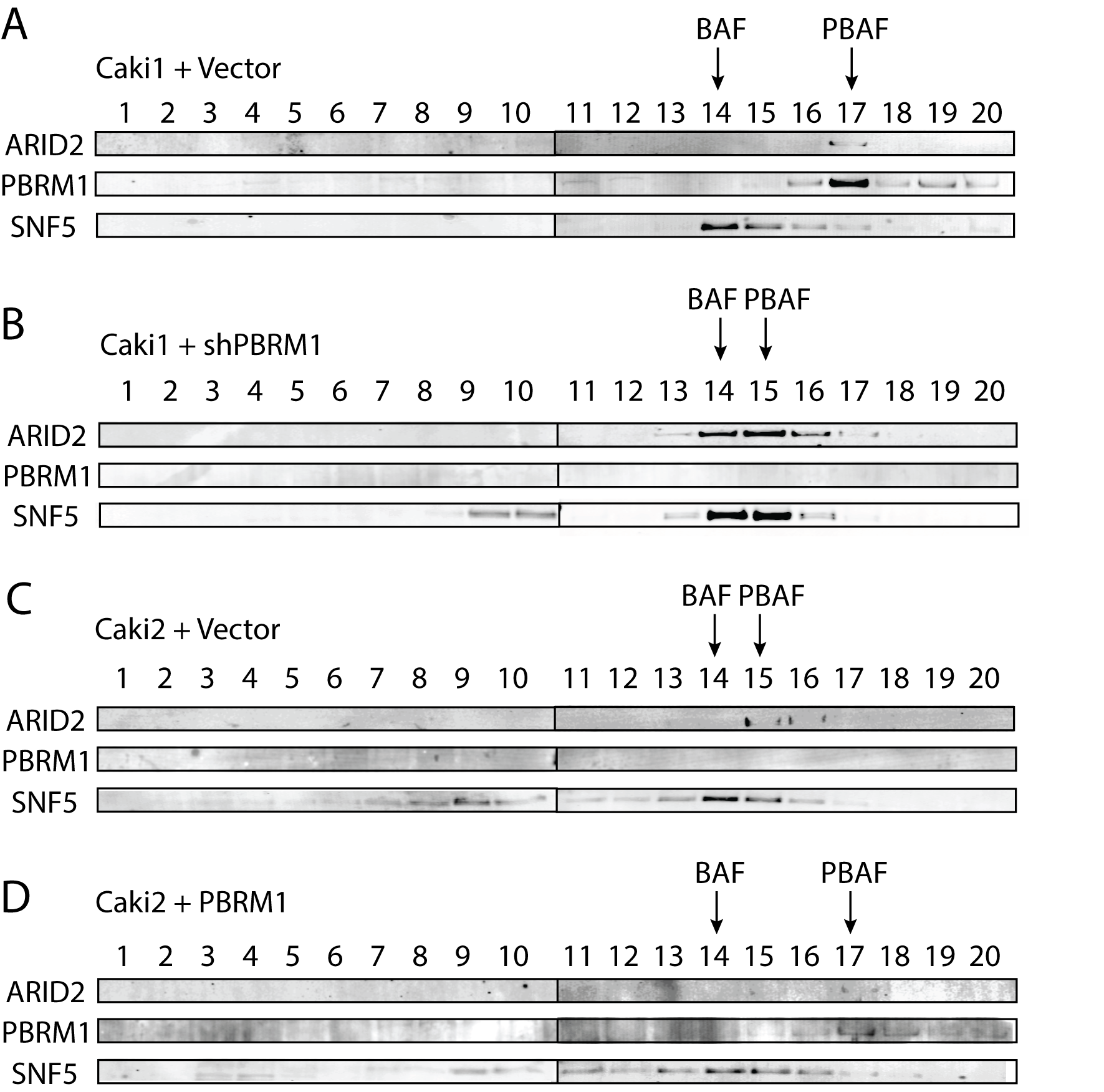

Supplement: S1 Fig — (A) In Caki1 cells PBAF (represented by PBRM1 and ARID2) elutes in higher fractions of a glycerol gradient indicating a larger size than BAF (represented by SNF5, which exists in both BAF and the less abundant PBAF), (B) Upon PBRM1 knockdown, the size of PBAF decreases, as represented by a shift in ARID2 staining to earlier fractions (C) In Caki2 cells with PBRM1 mutation, ARID2 staining is detected in an earlier fraction. (D) Upon the re-expression of PBRM1, the size of PBAF (represented by ARID2 staining) increases and elutes in later fractions, mimicking the staining profile observed for Caki1 cells. (TIF) [file pone.0153718.s001.tif]

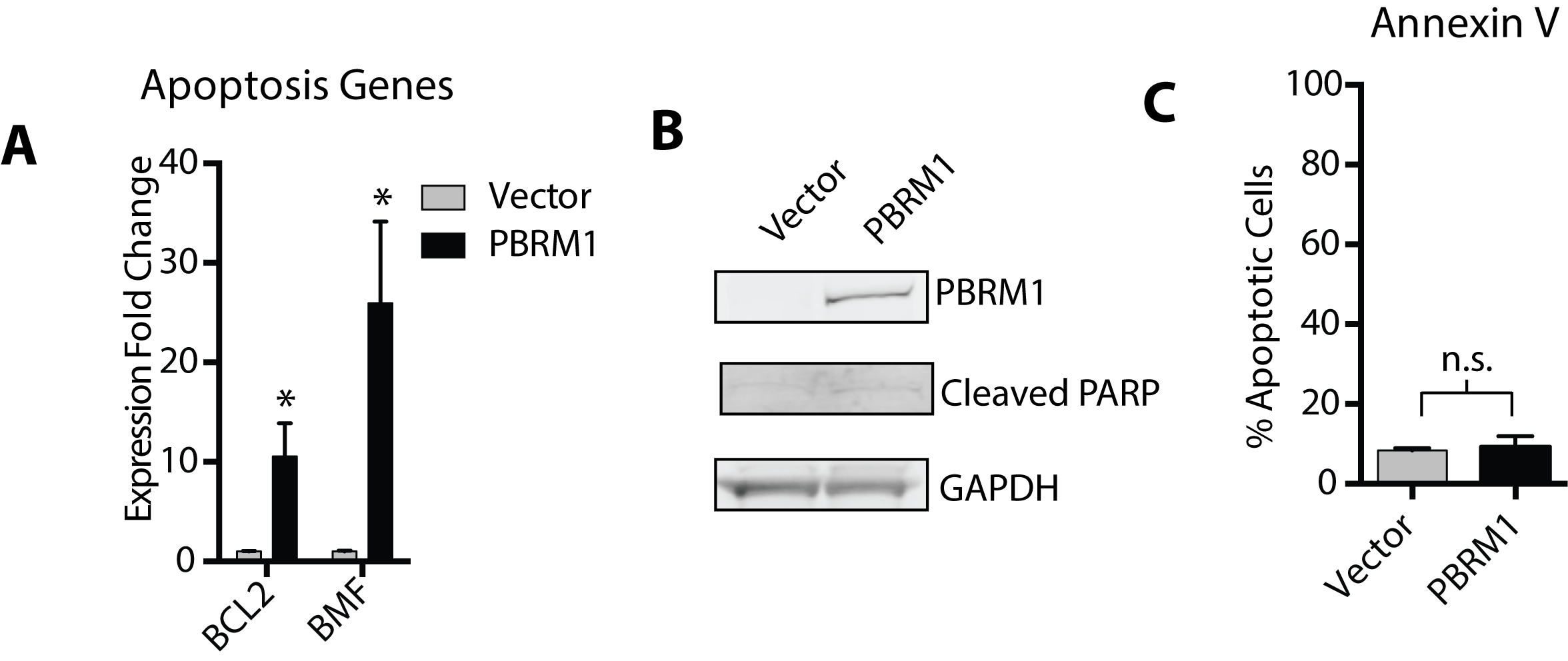

Supplement: S2 Fig — (A) Validating relative expression of genes regulating apoptosis (identified in RNA_seq data) in Caki2+Vector and Caki2+PBRM1 cells by qRTPCR. A designation of * indicates p < 0.05 (Student t-test). n = 3 independent biological replicate experiments. Error bars represent s.e.m. (B) Blot of cleaved PARP (apoptotic marker) and (C) Percentage of apoptotic cells determined by flow cytometric analysis on Caki2+Vectorand Caki2+PBRM1 cells. n = 3 independent biological replicates. Error bars represent s.e.m. (TIF) [file pone.0153718.s002.tif]

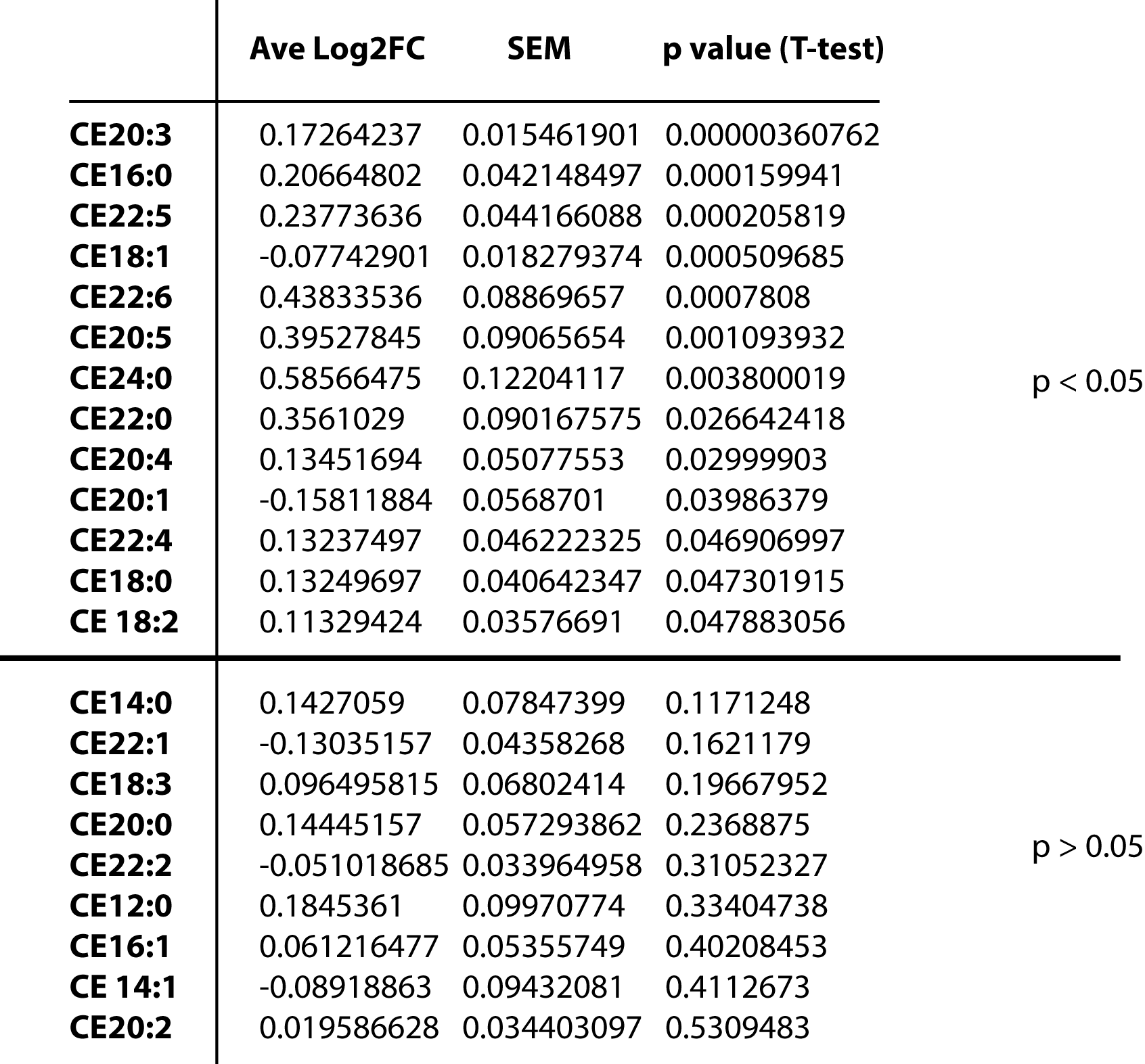

Supplement: S3 Fig — The log2 Fold change, SEM and p value (Student’s T-test) of the analyzed CEs. n = 3 biological replicates. (TIF) [file pone.0153718.s003.tif]

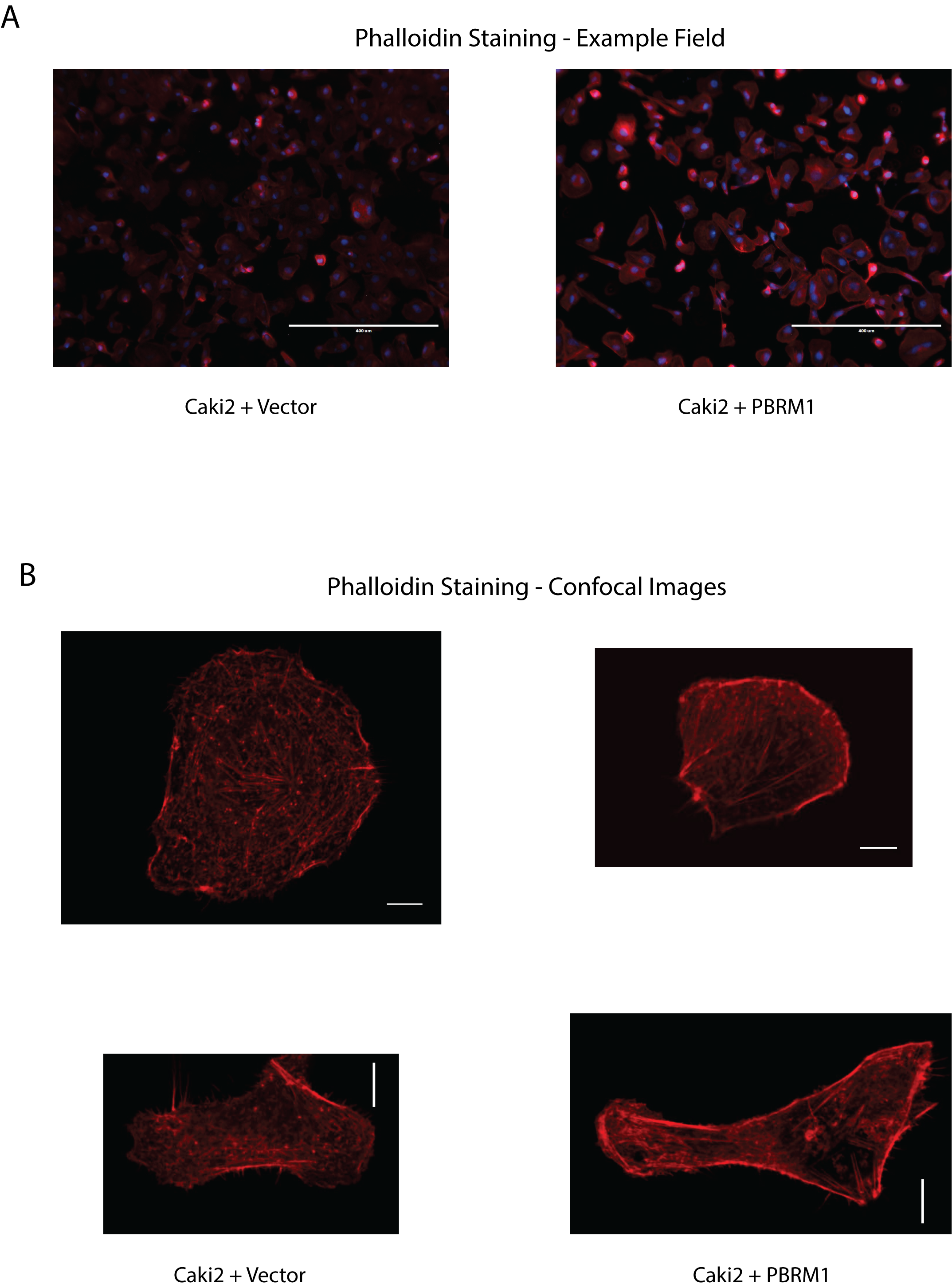

Supplement: S4 Fig — (A) Representative Field showing Phalloidin (F-actin) and DAPI (nucleus) staining in Caki2+Vector and Caki2+PBRM1 cells used for unbiased quantification of cells with high cortical actin. 4X magnified image of this is shown in Fig 6B. (B) Examples of confocal images of Caki2+Vector and Caki2+PBRM1 cells stained with Phalloidin. Scale bar depicts 10 µm. (TIF) [file pone.0153718.s004.tif]

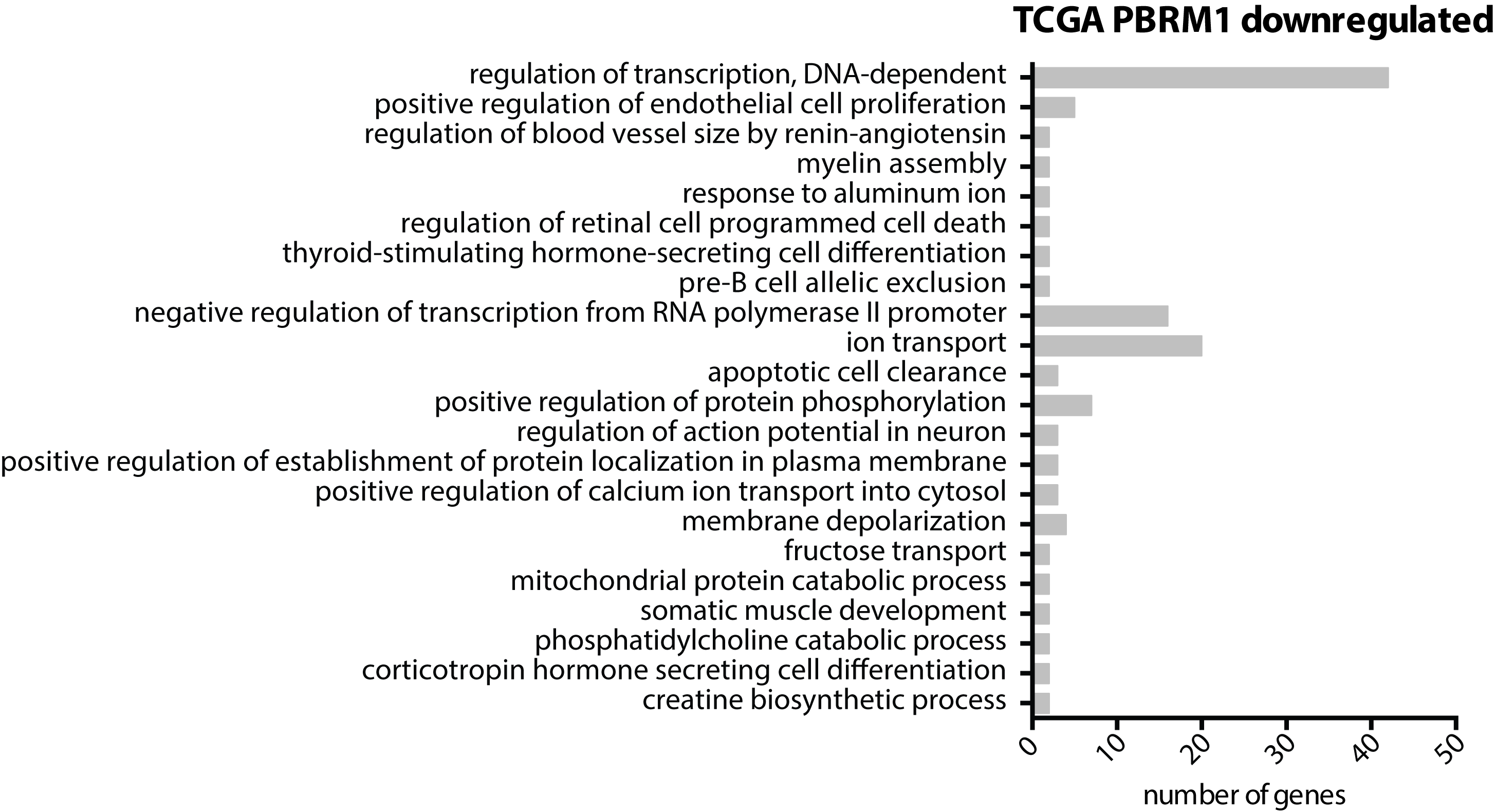

Supplement: S5 Fig — GO Biological Processes enriched by genes downregulated in TCGA ccRCC biospecimen with no PBRM1 mutation compared to ccRCC biospecimen with PBRM1 mutations. (TIF) [file pone.0153718.s005.tif]
